# Acute changes in extracellular volume fraction in skeletal muscle monitored by ^23^Na NMR spectroscopy

**DOI:** 10.14814/phy2.13380

**Published:** 2017-09-04

**Authors:** Teresa Gerhalter, Pierre G. Carlier, Benjamin Marty

**Affiliations:** ^1^ Institute of Myology NMR Laboratory Paris France; ^2^ CEA DRF IBFJ MIRCen NMR Laboratory Paris France

**Keywords:** Extracellular volume fraction, proton T2, skeletal muscle, sodium NMR

## Abstract

In this article, we induced acute changes in extracellular volume fraction in skeletal muscle tissue and compared the sensitivity of a standard ^1^H T_2_ imaging method with different ^23^Na‐NMR spectroscopy parameters within acquisition times compatible with clinical investigations. First, we analyzed the effect of a short ischemia on the sodium distribution in the skeletal muscle. Then, the lower leg of 21 healthy volunteers was scanned under different vascular filling conditions (vascular draining, filling, and normal condition) expected to modify exclusively the extracellular volume. The first experiment showed no change in the total sodium content during a 15 min ischemia, but the intracellular weighted ^23^Na signal slowly decreased. For the second part, significant variations of total sodium content, sodium distribution, and T_1_ and $T_{2}^{*}$ of ^23^Na signal were observed between different vascular filling conditions. The measured sodium distribution correlates significantly with sodium T_1_ and with the short and long $T_{2}^{*}$ fractions. In contrast, significant changes in the proton T_2_w signal were observed only in three muscles. Altogether, the mean T_2_w signal intensity of all muscles as well as their mean T_2_ did not vary significantly with the extracellular volume changes. In conclusion, at the expense of giving up spatial resolution, the proposed ^23^Na spectroscopic method proved to be more sensitive than standard ^1^H T_2_ approach to monitor acute extracellular compartment changes within muscle tissue.

## Introduction

Cellular life depends on the ability to tightly control the solute/water balance. Water not only passively distributes bidirectionally into the extra‐ and intracellular compartments, water channel proteins, so‐called aquaporin, also accelerate the water movement possessing a 10–100‐fold higher capacity for water permeation (Agre et al. 2002). This water flux directed by osmotic and hydraulic gradients is determined by the effective osmotic equilibrium and establishes cell volume. There is a variety of osmotically active molecules that are involved in the volume control mechanisms, of which the major ionic contributors are sodium, potassium, and chloride (Guyton and Hall 2006). The sodium ion distribution in biological tissues plays therefore a crucial role in cellular function and homeostasis, and is closely linked to changes in extracellular volume fraction. Na^+^ has also an essential role in the energy‐consuming processes of membrane transport by the sodium–potassium ATPase. While intracellular Na^+^ concentrations remain at a low level of about 10–15 mmol/L, extracellular concentrations range between 140 and 150 mmol/L (Skou 1998). This ion transport performed against the electrochemical gradient is essential to protect the cell from bursting as a result of osmotic swelling. Impairments of the energy metabolism or disruption of the cell membrane integrity causes a loss of Na^+^ homeostasis and thus an increase in the intracellular sodium concentration that can lead ultimately to cell death. As a result, changes in sodium intracellular concentration can be expected in disorders altering cell function/integrity, including several neuromuscular disorders (NMD) (Dunn et al. 1993; Jurkat‐Rott et al. 2010).

While extracellular concentrations can be easily measured with blood sampling, noninvasive techniques capable to assess changes in the extracellular volume fraction or intracellular Na^+^ concentration are desired to study ionic homeostasis, the still many unraveled facets of it, and to better understand alteration by diseases. NMR‐based methods are essentially noninvasive and can provide useful information on the extracellular volume fraction in healthy and diseased subjects. Numerous NMR techniques have been proposed for measuring cell volume fraction including water diffusion and *T*_2_ weighted sequences (Moseley et al. 1990) or using an extracellular contrast agent like Gd‐DTPA (Mulkern et al. 1989).

As a supplementary method to the classical ^1^H‐NMR, ^23^Na‐NMR lends itself as a unique noninvasive tool for in vivo investigation of ionic homeostasis in the human body. Alterations in the ^23^Na signal have been already observed in various pathologies such as tumors, stroke, myocardial infarction, and myopathies. However, the central question for clinical applications is whether ^23^Na MR can deliver additional information, which cannot be obtained or at least cannot be more conveniently accessed by other means, including ^1^H‐NMR.

Currently, two ^23^Na‐NMR methods are used in vivo in human studies to separate between intra‐ and extracellular ^23^Na signal. One method utilizes differences of ^23^Na longitudinal relaxation rates in different physiological compartments and selectively suppress one component via inversion‐recovery (IR) sequences (Nagel et al. 2011). In this approach, only one type of environment with one specific recovery rate of the ^23^Na longitudinal magnetization can be suppressed. Regions with similar recovery rates might be also unintentionally suppressed. The other method is based on the 3/2 spin, a specific feature of the sodium nuclei, leading to quadrupolar interactions. Sequences based on triple quantum filtering (TQF) may be employed to specifically select the NMR signal of sodium ions being restricted in their mobility. This restriction is caused by interactions with macromolecular structures, located preferentially within the intracellular compartment (Payne and Styles 1991). The TQF as well as the IR technique cannot completely separate the intracellular from the extracellular compartment due to the complex distribution of the spectrum types and relaxation times. However, they allow a signal weighting towards the intracellular compartment and therefore generate useful information on cell viability. The residual quadrupolar interactions observed in restricted biological tissues also result in a biexponential transverse relaxation behavior with a short T_2_ of the order of 0.5–5 msec and a long T_2_ of the order of 10–30 msec. This is typically observed in the intracellular space, while the extracellular space, where the ions are more freely tumbling, is generally associated with a monoexponential T_2_ decay.

In this study, we propose a multiparametric approach using nonlocalized ^23^Na‐NMR spectroscopy to produce quantitative indices reflecting the sodium distribution in the skeletal muscle, in particular the intracellular sodium content and the extracellular volume fraction. We considered that we would achieve a more relevant characterization of the Na^+^ distribution and cellular interactions by giving up the spatial information and by focusing more on the ^23^Na NMR properties in the skeletal muscle.

In order to test the validity of these approaches, and in particular the ability of ^23^Na spectroscopy to detect and monitor changes in Na^+^ distribution between intra‐ and extracellular compartments, we performed the experiments reported here. As a first step, we monitored the effect of a short‐term ischemia on the Na^+^ biodistribution imposed to the leg skeletal muscle tissue. Then, we performed measurements on the same lower leg muscles under different vascular filling conditions to manipulate the ratio of intra/extracellular contributions to the Na^+^ pool and to test our ability to detect it by multiparametric ^23^Na‐NMR. We compared the sensitivity of three short‐acquisition‐time nonlocalized ^23^Na‐NMR sequences to that of a standard ^1^H T_2_ imaging method to monitor varying extracellular volume fractions conditions.

## Methods

### Subjects and study design

Sodium NMRS and proton NMRI were performed on 28 healthy volunteers (aged 28.2 ± 8.4 years, 13 women and 15 men) as part of a methodology protocol approved by the local ethics committee (Comité de Protection des Personnes Ile de France VI). Before data acquisition, written informed consent was obtained from all subjects. Subjects underwent one of the two protocols described below. The first group of seven volunteers participated in protocol 1 dedicated to investigate the effect of a short‐term ischemia on the sodium distribution in the skeletal muscle (group 1). The other 21 volunteers were assigned to group 2 (*n* = 14) and group 3 (*n* = 7), which underwent protocol 2 with either ^23^Na‐NMR (group 2) or ^1^H T_2_ (group 3) acquisitions to compare the sensitivities of the two methods to detect acute changes in intra/extracellular volume fractions.

#### Protocol 1

**Figure 1:**
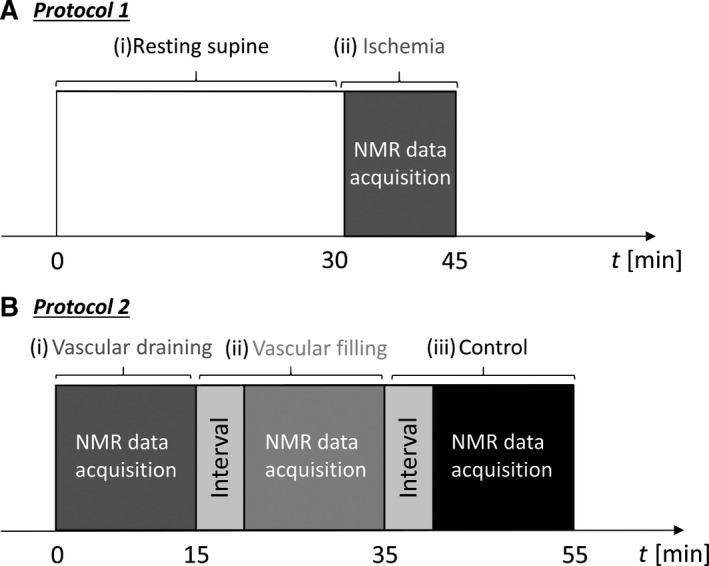
Timeline of two protocols including different vascular filling conditions. (A) Protocol 1: (i) After 30 min supine rest; (ii) the medical cuff placed above the knee was rapidly inflated and the acquisition of the sodium spectra started. (B) Protocol 2: (i) vascular draining was first applied with the calf positioned in the coil followed by the NMR acquisition (nonlocalized ^23^Na spectroscopic and ^1^H imaging acquisition or by ^1^H MSME acquisition). (ii) After vascular filling during a 5 min interval initiated by a reduced cuff pressure, another NMR acquisition was launched. (iii) Normal condition: the pressure of the cuff was completely released and after a 5 min break the final sodium NMR and proton imaging were performed.

^23^Na‐NMR spectra were acquired during a short ischemia in the right calf of group 1 (Fig. 1A). Prior to the NMR acquisition, a medical cuff was placed above the knee and the subjects rested supine on the bed during 30 min. Then, the cuff was connected to a circuitry pressurized at 250 mmHg to block instantaneously and completely the arterial inflow and the venous outflow. The cuff pressure was manually controlled from the outside of the magnet room by the manipulator using a mercury manometer (MERCUREX+; Spengler, Antony, France) combined with a fast switch valve system. During the 15 min ischemia, sodium homeostasis was monitored with series of short ^23^Na sequences.

#### Protocol 2

^23^Na‐NMR spectra and ^1^H‐NMR images of groups 2 and 3 were acquired under three different vascular filling conditions: (1) vascular draining, (2) vascular filling, and (3) normal condition. The protocol timeline for the data acquisition is presented in Figure 1B. These vascular conditions were imposed while the subject remained still in supine position inside the scanner. Vascular draining was initiated by wrapping an elastic compression band from toes to the knee. Then, a medical cuff, placed over the knee, was inflated at 250 mmHg to block completely the arterial inflow and the venous outflow. The elastic compression band was then removed from the leg while keeping the cuff pressure high and after centering the leg in the magnet the first set of data was acquired. Next, the cuff pressure was decreased to 60–70 mmHg in order to fill the capacitance vessels. After a 5 min interval to allow for a stabilized vascular filling condition, another NMR data acquisition was performed. Finally, the cuff was fully released and again 5 min were given, this time, to resume normal perfusion regime and the last series of NMR sequences was acquired.

### NMR protocol

All NMR experiments were performed on a 3T whole body scanner (PRISMA; SIEMENS Healthcare, Erlangen, Germany).

#### ^23^Na‐NMR protocol

For groups 1 and 2, ^23^Na NMRS sequences were acquired with a custom‐built linear volume ^23^Na leg coil (inner diameter 18 cm with length of 16 cm). The center of the coil was installed at the biggest circumference of the leg. The length of the coil is 20 cm, which allows covering the biggest part of the leg muscles. To minimize the leg movement, foam pads were inserted between the leg and the coil. For each subject, the coil was tuned manually prior to experiments. A B_1_^+^ calibration sequence using rectangular pulses determined the reference voltage for a 90° excitation pulse. This voltage was then applied as the reference voltage for the entire ^23^Na‐NMR protocol.

The ^23^Na spectroscopy part consisted in free induction decay (FID), triple quantum filtering (TQF), and inversion‐recovery Look‐Locker (IR‐LL) sequences with a total acquisition time of 14 min for the second protocol. The FID sequence, allowing the quantification of total sodium content, was acquired with the following parameters: TR = 300 msec, number of averages (NEX) = 400, time delay = 200 *μ*sec, receiver bandwidth (BW) = 2 kHz, vector size = 128 pts, T_Acq_ = 2 min. The TQF acquisition, assessing the fraction of sodium bound to macromolecules or in fast exchange with them, was realized by a 6‐steps‐phase cycling scheme (Benkhedah et al. 2013) with three 90° excitation pulses of different phases to filter only triple quantum transitions (*ϕ*_1_ = 30°, 90°,150°,210°,270°,330°; *ϕ*_2_ = *ϕ*_1_ + 90°; *ϕ*_3_ = 0°; preparation time *τ*_1_ = 11 msec, evolution time *τ*_2_ = 200 *μ*sec, and *τ*_3_ = 350 *μ*sec, TR = 100 msec, NEX = 3000, BW = 2 kHz, vector size = 128 pts, T_Acq_ = 5 min). To measure the global ^23^Na longitudinal relaxation time, T_1_, the IR‐LL sequence was acquired with 75 different inversion times (from 5 to 375 msec), flip angle (FA) = 3°, TR = 450 msec, NEX = 950, BW = 10 kHz, vector size = 32 pts, and T_Acq_ = 7 min.

For group 1, the number of averages for the FID and TQF were reduced to acquire the two sequences with a temporal resolution of 1 min. The FID had 40 averages (T_Acq_ = 12 sec) and the TQF 500 averages (T_Acq_ = 50 sec).

Low‐resolution ^1^H imaging was added to the ^23^Na‐NMR protocol part using the body coil for magnetization excitation as well as signal reception. Changes in the leg volume between the different vascular filling conditions were monitored with a 3D FLASH sequence covering the whole calf. This sequence was acquired with the following parameters: TE/TR = 2.78/65 msec, matrix size = 250 × 250 × 50 mm^3^, spatial resolution = 2.6 × 2.6 × 5 mm^3^, and T_Acq_ = 1 min.

#### ^1^H‐NMR protocol

**Figure 2:**
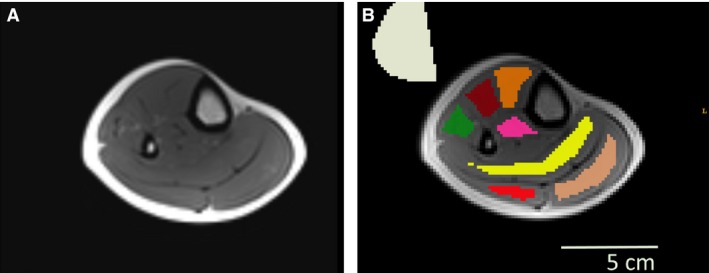
Standard ^1^H T_2_ imaging: (A) a cross‐sectional T_2_w image (TE = 34 msec); (B) The regions of interest used for analysis were traced within the muscles (TA orange; ED brown; TP pink; PL green; SO yellow; GL red; GM beige; Background noise gray).

For group 3, proton images were acquired with a 15 channel ^1^H knee coil (Fig. 2A). Foam pads were also inserted between the leg and the coil to minimize leg movement. To measure proton T_2_‐weighted signal intensities and proton T_2_ values, a multiple‐slice multiple‐echo (MSME) sequence was acquired with 17 different echo times (from 8.5 ms to 144.5 ms), TR = 3000 msec, 5 slices with matrix size = 192 × 192 × 10 mm^3^, spatial resolution = 1.5 × 1.5 × 10 mm^3^, and T_acq_ = 3 min.

### Analysis of ^23^Na‐ and ^1^H‐NMR data

Sodium data were processed offline with MATLAB (MathWorks, Natick, MA). After reconstruction of ^23^Na spectra, the area under the curve was calculated from the FID and the TQF spectra to derive the FID signal, the TQF signal, and thus the TQF/FID ratio. In contrast to the FID, which presents the total sodium signal, the TQF signal is weighted by the intracellular sodium. The TQF/FID ratio thus reflects the sodium biodistribution between the intra‐ and extracellular space. In addition, $T_{2}^{*}$ spectra were obtained from the temporal decay of the FID by the deconvolution method presented in the study by Araujo et al. (2014). This allowed to determine a short $T_{2}^{*}$ fraction as well as short and long $T_{2}^{*}$ values for Na^+^. A global T_1_ relaxation time was estimated from the IR‐LL sequence by a monoexponential adjustment of the signal recovery at different time points after magnetization inversion.

Total muscle volume estimation was performed on ImageJ (Rasband 2017) from the ^1^H FLASH images. Regions of interest (ROIs) were manually drawn around the leg on 23 slices. Leg volumes were then calculated based on the exported ROIs (leg area × slice thickness).

Proton T_2_‐weighted signal intensities and proton T_2_ values were measured from the MSME sequences with Python codes. ROIs were manually traced inside specific muscles (see Fig. 2B; ED extensor digitorum, GL gastrocnemius lateral, GM gastrocnemius medial, PL peroneus longus, SO soleus, TA tibialis anterior, TP tibialis posterior). Proton T_2_‐weighted (T_2_w) signal intensities were calculated as a mean signal intensity of each ROI in the T_2_w image at TE of 34 msec and the T_2_ maps were produced via a standard monoexponential fit (De Graaf 2007).

### Statistical analysis

Data were analyzed and expressed as mean and standard deviation. Percentages of change of each parameter as compared to the control condition were determined. Statistical analysis was performed in SPSS (SPSS 22, SPSS Inc. Chicago, IL) using ANOVA with repeated measures and Bonferroni post hoc tests for pairwise comparisons to evaluate significant differences between the different conditions. Pearson correlations analyzed the relationship between the variables. In all statistical tests, *P* < 0.05 was considered statistically significant.

## Results

### Impact of ischemia on ^23^Na‐NMR signal

**Figure 3:**
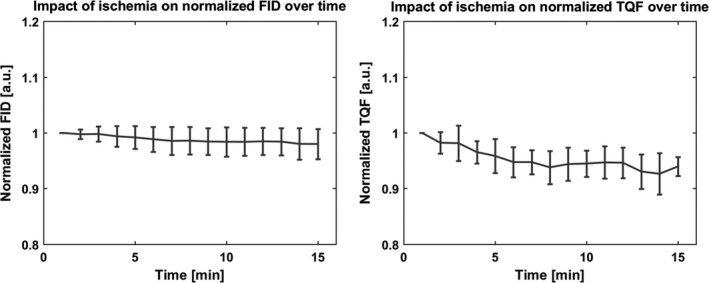
The effect of short‐term ischemia in vivo in the skeletal muscle measured by ^23^Na NMR on seven healthy volunteers.

Figure 3 shows the evolution of the FID and TQF signals during the 15 min ischemic period. The signals have been normalized using the first time point as a reference and are reported as mean values and standard deviation. The FID signal changes with time by 1.8 ± 2.4%, not significant with repeated measures ANOVA. The TQF signal was found to progressively decrease over time (−5.8 ± 2.9% at the end of the experiment). Repeated measures ANOVA indicated a significant decrease in the TQF signal after 15 min of ischemia (*P* < 0.05).

### Vascular filling changes monitored by ^23^Na‐NMR

**Figure 4:**
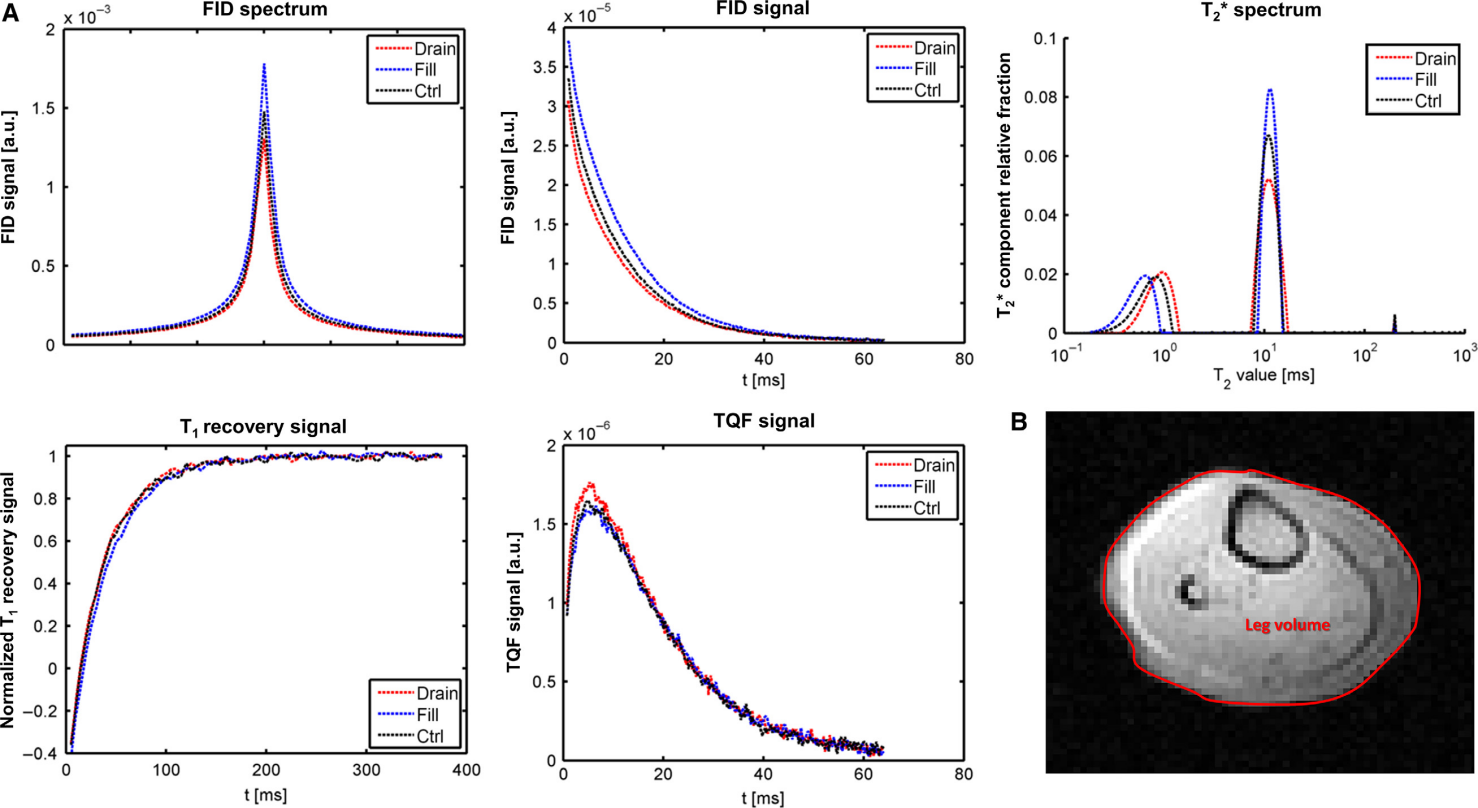
The sodium FID, TQF, and T_1_ recovery signals measured in a healthy young 22‐year‐old female volunteer under different vascular filling conditions. (A) The FID signal in the time domain is Fourier transformed to obtain the FID spectrum, which gives then the $T_{2}^{*}$ spectrum by a $T_{2}^{*}$ deconvolution of the FID signal decay. The $T_{2}^{*}$ spectrum is plotted with logarithmically scaled abscise. The plots show the characteristic course of the vascular draining (red), vascular filling (blue) and control (black) conditions. (B) The drawn region of interest on the FLASH image of the right calf of the volunteer were used for the volume calculations. FID, free induction decay; TQF triple quantum filtering.

Figure 4A displays the different sodium indices obtained by ^23^Na‐NMR in one volunteer of the group 2 under each vascular filling condition. As depicted, the FID, TQF signal, and T_1_ relaxation curves were modified in response to extracellular volume changes. The FID signal, expressed as the area under the peak in arbitrary units, decreased from 8.2 × 10^−3^ for the control condition to 7.4 × 10^−3^ while draining and increased to 9.7 × 10^−3^ under vascular filling. The TQF signal, also expressed as the area under the peak in arbitrary units, showed a different behavior with a decrease from 5.5 × 10^−5^ to 5.4 × 10^−5^ during vascular filling and increases to 5.8 × 10^−5^ during vascular draining. The T_1_ relaxation time showed a parallel behavior as the FID signal, by dropping from 34.3 to 33.7 msec during draining and raised to 37.0 msec during filling. After deconvolution, two well resolved Na^+^ pools could be identified for each vascular filling condition on the $T_{2}^{*}$ spectra. One slice of the ^1^H FLASH volume is also presented in Figure 4B, with the manual delineation of the lower leg. For this subject, the leg muscle volume was 1175 cm^3^ during vascular draining, 1273 cm^3^ during vascular filling, and 1180 cm^3^ under control conditions.

**Figure 5:**
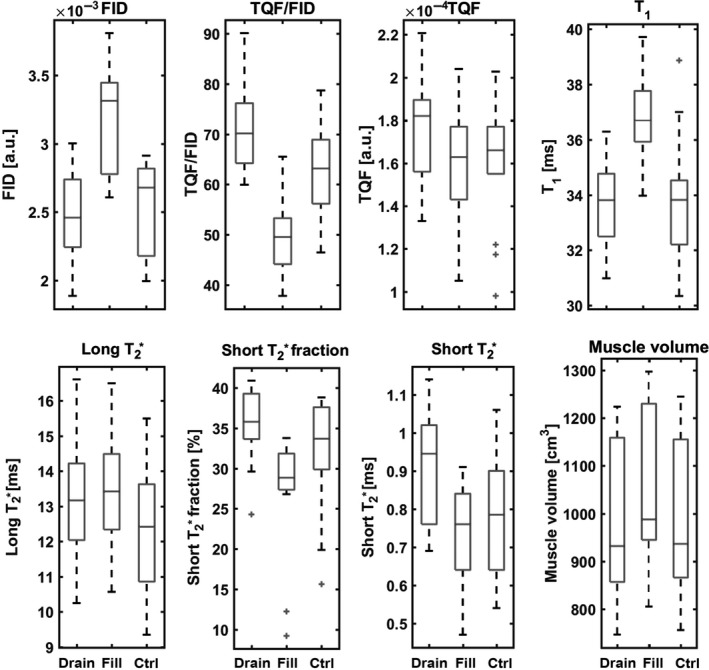
Evolution of different ^23^Na parameters (FID signal, TQF/FID ratio, TQF signal, T_1_ value, long $T_{2}^{*}$ signal, short $T_{2}^{*}$ fraction, and signal) and leg volume under three different vascular filling conditions (vascular draining: drain, vascular filling: fill, and control condition: ctrl). The boxes have lines at the lower quartile, median, and upper quartile with the length of the whiskers specified as 1.5 times the interquartile range. The crosses mark outliers of the datasets. FID, free induction decay; TQF, triple quantum filtering.

Figure 5 summarizes the mean values of the different indices extracted from the ^23^Na spectroscopic sequences and the leg volume of all subjects under the three different vascular filling conditions. Repeated measures ANOVA tests revealed significant variations of all the parameters between the three conditions (*P* < 0.05). At rest, the mean sodium FID signal was 2.5 ± 0.3 × 10^−3 ^a.u., the global sodium T_1_ was 33.8 ± 2.2 msec, the sodium TQF signal 1.6 ± 0.3 × 10^−4 ^a.u and the TQF/FID ration 6.3 ± 1.0 × 10^−2^. Based on the $T_{2}^{*}$ deconvolution, mean skeletal muscle tissue sodium short $T_{2}^{*}$ and long $T_{2}^{*}$ were $T_{s,2}^{*}$ = 0.8 ± 0.2 msec and $T_{1,2}^{*}$ = 12.4 ± 1.8 msec with mean fraction of 32 ± 7 and 68 ± 7%, respectively. For all subjects, the mean leg volume at rest was 995.7 ± 160.2 cm^3^.

All measured changes of the ^23^Na variables and leg volume were calculated as percentage of change between the vascular filling or draining condition and the control condition.

The ^23^Na FID signal was not significantly decreased under the drainage condition (−3.8 ± 7.4%) and significantly increased in the filling conditions (+22.7 ± 3.2%; *P* < 0.05), compared with the control condition. The opposite trend was observed for the short $T_{2}^{*}$ fraction and the TQF/FID ratio, which both raised significantly during vascular draining (+13.0 ± 15.3 and +13.1 ± 6.1%, respectively; both *P* < 0.05) and significantly decreased with vascular filling as compared to normal conditions (−18.2 ± 23.0 and −23.4 ± 4.9%, respectively; both *P* < 0.05). In parallel, the long $T_{2}^{*}$ value was significantly increased during draining and filling (+6.4 ± 5.6%; *P* < 0.05, +7.9 ± 4%; *P* < 0.05, respectively). The short $T_{2}^{*}$ value increased significantly during vascular draining (+18.0 ± 14.7%; *P* < 0.05) and tends to decrease during the filling condition (−6.4 ± 9.8%). TQF signal was significantly higher with vascular draining (+9.3 ± 9.9%; p < 0.05) and did not vary during vascular filling (−0.7 ± 5.7%). On the contrary, significant increase was observed for global T_1_ values and muscle volume during vascular filling (respectively, +8.2 ± 3.1 and +5.5 ± 2.0%; *P* < 0.05) while no significant alterations were detected during vascular draining (respectively, −0.2 ± 3.5 and −1.7 ± 1.8%).

**Figure 6:**
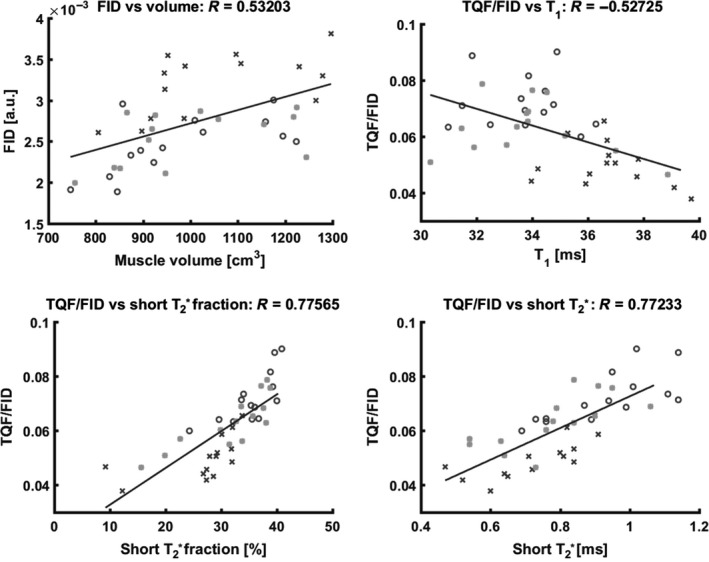
Correlation of different ^23^Na parameters and leg volume. Correlation between FID signal and volume and between TQF/FID ratio and T_1_ value. Additionally, highly significant correlations between TQF/FID ration and short $T_{2}^{*}$ fraction and short $T_{2}^{*}$ values were detected. Data acquired under vascular draining and vascular filling are depicted as circles and crosses, respectively. Asterisks represent the data points collected during the control condition. FID, free induction decay; TQF, triple quantum filtering.

The changes in the FID signal and in muscle volume correlated significantly (*R* = 0.53), whereas no significant correlation was observed between the TQF signal changes and the muscle volume (*R* = 0.17). Significant linear dependences between the TQF/FID ratio and T_1_ values (*R* = −0.53) and all the $T_{2}^{*}$ parameters (short $T_{2}^{*}$ fractions: *R* = 0.78; short $T_{2}^{.8*}$: *R* = 0.77; and long $T_{2}^{.5*}$: *R* = 0.52) were detected. A selection of correlations from different ^23^Na parameters and the leg volume are depicted in Figure 6.

### Vascular filling changes monitored by ^1^H‐NMR

The same vascular filling paradigm was applied to investigate the sensitivity of ^1^H T_2_ imaging to detect the extracellular volume fraction changes. Proton T_2_ values and proton T_2_w intensities were measured for different muscles, from which a mean value for all muscles was determined. At rest, the mean T_2_ was 43.3 ± 1.7 msec and the mean T_2_w intensity was 547.3 ± 16.3 a.u. Besides, all measured changes of the ^1^H T_2_ and ^1^H T_2_w signal intensities were calculated as percentage of change between the vascular filling or draining condition and the control condition. Only two muscles changed significantly their T_2_ during the three conditions as determined by one‐way ANOVA (*P* < 0.05). Post‐hoc Bonferroni test disclosed that the T_2_ augmented significantly in the ED and the TP muscles during vascular filling compared to the control condition (1.7 ± 1.2 and 1.6 ± 0.9%, respectively). Furthermore, repeated measures ANOVA tests revealed significant variations of the T_2_w intensities between the three conditions for three muscles (ED, Gl, and TA; *P* < 0.05). Post hoc tests using the Bonferroni correction revealed that the T_2_w intensities of the ED and TA muscles increased significantly during vascular filling (3.8 ± 1.8 and 4.8 ± 1.4%, respectively) compared to the control condition, whereas the T_2_w intensity of the GL muscle increased significantly during vascular draining (4.2 ± 2.5%; *P* < 0.05) and showed no significant variation during the filling condition (1.0 ± 1.7%; *P* = 0.5).

**Figure 7:**
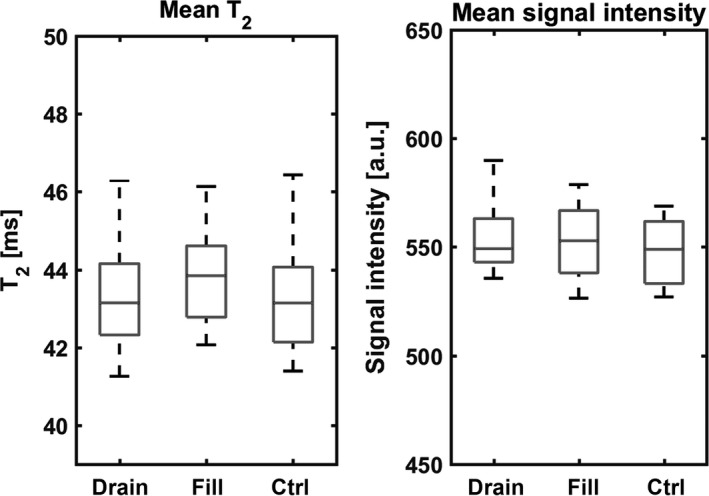
Evolution of the mean T_2_w signal intensities and the mean T_2_ for all muscles under the vascular filling and vascular draining conditions (vascular draining: drain, vascular filling: fill, and control condition: ctrl).

The mean T_2_ and the mean T_2_w intensity taken for all muscles did not significantly differ between vascular filling conditions (one‐way ANOVA test, Figure 7).

## Discussion

The intent of this study was to assess whether ^23^Na‐NMR could monitor acute changes in the extracellular volume fraction and to compare the sensitivity of this method to standard proton T_2_ imaging.

The compartmental exchange between intracellular and extracellular compartments and thus the cell volume fraction is regulated by osmotic and hydrostatic pressures. Sodium holds a central position in the homeostasis as it accounts for 90–95% of all solutes in the extracellular fluid while being the only cation exerting significant osmotic pressure (Marieb and Hoehn 2007). The idea of performing in vivo ^23^Na spectroscopy to assess homeostasis and cell viability in vivo is not novel and dates back to the 1980s (Blum et al. 1988; Eleff et al. 1988). However, the spectroscopic approach seems to be nowadays left aside by most research groups currently focusing their efforts on improving spatial resolution of ^23^Na images by taking advantage of high‐field MRI (Nielles‐Vallespin et al. 2007; Madelin et al. 2014; Linz et al. 2015). In this study, we showed that biophysically relevant information related to the physiological status of skeletal muscle tissue can be acquired in <15 min, at the expense of losing the spatial information. As a result of the high signal‐to‐noise ratio (SNR) of this spectroscopic approach, we were able to monitor several sodium NMR variables while filling or draining the vascular compartment. On the other hand, the standard proton signal intensities and the proton T_2_, which are usually used to characterize disease activity (inflammation, necrosis, and edema) have been found less sensitive to the extracellular volume fraction alterations when compared to the sodium parameters.

### 15 min ischemia

In the first part of our study, we investigated the effect of a short‐term ischemia on the sodium distribution in the human skeletal muscle. This was an important preliminary step to make sure that the interpretation of the ^23^Na signal changes during the vascular volume manipulations would not be complicated by concurrent alterations of the intracellular Na^+^ distribution. Contrary to earlier findings in the ischemic heart (Pike et al. 1990), the total sodium content did not significantly vary within this short period of blood flow interruption. This can easily be understood considering the huge energy demand difference between the two organs at baseline. However, the TQF within skeletal muscle tissue decreased by approximately 6% after the 15 min ischemia.

Babsky et al. (2008) studied the effect of prolonged global ischemia on Na^+^ concentrations in the rat skeletal muscle over 4 hours. In their work, the total Na^+^ measured by a FID was unchanged compared to the preischemic level during the first 2 hours of ischemia followed by a significant increase afterwards by 8–16%. In a second group of rats, they acquired the Na^+^ signal using a shift reagent to separate the intra‐ from the extracellular ^23^Na signals. Ischemia caused a progressive increase in the intracellular sodium concentration ([Na^+^]_i_) but only after the first hour of ischemia, with a parallel decrease in the extracellular sodium concentration ([Na^+^]_e_). In the rat heart during cardioplegic arrest, it was reported that [Na^+^]_i_ increased during ischemia only after an initial delay of 15 min (Schepkin et al. 1998).

The same ischemic protocol was used before in our laboratory to study its impact on muscle oxygenation and perfusion. The ADP concentration increased by 7% compared to the resting state after 6 min of ischemia indicating already a slight impairment of the muscle ATP metabolism (Brillault‐Salvat et al. 1997). A reduced activity of the Na^+^–K^+^ ATPase would result in an increased intracellular sodium content as well as cell swelling. Regarding ^23^Na nucleus, this effect would translate in an increase of the TQF signal (Dizon et al. 1996; Tauskela et al. 1997). In our situation, the small decrease in TQF signal that was observed during the 15 min ischemia is contraintuitive at first sight. Nevertheless, a slight expansion of the intracellular space or a decrease in water exchange rate between the different compartments might also decrease sodium‐protein interactions in this compartment, and consequently, decrease anisotropic quadrupolar interactions and the TQF signal. This interesting feature will be investigated in a future protocol on animal models using a shift reagent, where this ischemic paradigm could be prolonged after 15 min.

### Extracellular volume fraction changes

We then investigated the sensitivity of nonlocalized ^23^Na spectroscopy to monitor acute changes in the extracellular volume fraction including vascular draining and vascular filling. We hypothesized that the rapid inflation of the air cuff above the knee to 250 mmHg does instantaneously stop the blood circulation, and has no further high impact on the [Na^+^]_i_ during our protocol. We considered the alterations of the ^23^Na signals being mainly due to changes of the extracellular volume fractions, or modification of interactions with macromolecules within the skeletal muscle tissue.

First, although the FID signal sums extra‐ and intracellular sodium content, changes in the extracellular volume could clearly be detected with this sequence. To our knowledge only one group before us has performed a ^23^Na NMRS study of the human skeletal muscle during short ischemic periods. Binzoni et al. (1998) analyzed the interstitial fluid displacement in the gastrocnemius during short ischemic cycles. In their study, FID signal decreased during ischemia, followed by a rapid sodium increase during the hyperemic reperfusion phase, which they attributed to volume changes in extracellular fluids. In our work, we confirmed this hypothesis by the significant correlation observed between the muscle volume estimated with ^1^H imaging and the ^23^Na FID signal.

Besides, slowly tumbling Na^+^ content in the muscle tissue was measured by acquisition of triple‐quantum‐filtered spectra. Theoretically, as the TQF preparation filters out first‐ and second‐order coherences, only ^23^Na experiencing quadrupolar interactions are highlighted. The TQF signal is often associated to the sodium pool bound to macromolecules, which is mainly found in the intracellular matrix. Changes in TQF signal are thus interpreted as modifications of intracellular Na^+^ concentration or intracellular volume fraction. However, some studies have already demonstrated that the ^23^Na TQF signal has contributions from the intra‐ as well as the extracellular Na^+^ pool. In previous work, the proportion of the total TQF signal originating from the extracellular compartment was found to be between 30% and 60% of total TQF signal (Schepkin et al. 1998; Navon et al. 2001; Eykyn et al. 2015). The TQF arising from the extracellular compartment is likely a result from ^23^Na nuclei being in close interaction with the macromolecules, which could explain the large correlation between TQF signal variations and intracellular Na^+^ content generally reported. Our results identified a small but significant increase in the TQF signal under vascular draining as compared to control condition (+9.3 ± 9.9%), while no significant changes were noticed between vascular filling and control condition. This observation tends to confirm the hypothesis that part of the TQF signal reflects the ^23^Na nuclei within the interstitial space, which is a priori the only compartment experiencing large modifications during our physiological paradigm. The increase in TQF signal likely reflects a more important interaction between ^23^Na nuclei and interstitial macromolecules caused by the additional drainage by the elastic band.

Longitudinal and transverse relaxation time variations were evaluated during this protocol. Monoexponential T_1_ recovery and biexponential $T_{2}^{*}$ decay behaviors were observed in the calf muscle tissues. This corresponds to what is generally observed in the skeletal muscle (Winter and Bansal 2001), and is explained by a two‐component model in fast‐exchange regime with a pool of Na^+^ ions within bulk isotropic medium and a pool of Na^+^ ions bound to macromolecules experiencing quadrupolar interactions. Global ^23^Na T_1_ relaxation times were significantly increased during vascular filling but this parameter did not allow to differentiate between vascular draining and control conditions. This parameter was not the most sensitive index to discriminate between the three vascular filling conditions of our physiological paradigm.

Based on the $T_{2}^{*}$ deconvolution of the FID signal decay, mean $T_{2}^{*}$ skeletal muscle tissue sodium relaxation times at rest were $T_{s,2}^{*}$ = 0.8 ± 0.2 msec (mean fraction of about 32%) and $T_{1,2}^{*}$ = 12.4 ± 1.8 msec. These results are in agreement with previous findings. For example, at 1.5 T in the skeletal muscle, Constantinides et al. (2000) found short and long $T_{2}^{*}$ equal to 0.5 ± 2.1 and 12.3 ± 1.9 msec, respectively. The short $T_{2}^{*}$ fraction was increased during draining, decreased during vascular filling, and correlated significantly with the TQF/FID ratio. This confirms that the variation of the Na^+^ content bound to macromolecules and experiencing quadrupolar interactions could be efficiently monitored with our spectroscopic protocol. Finally, the TQF/FID ratio was found to be the most robust and sensitive index to discriminate between the three conditions.

### Comparison of ^23^Na and ^1^H NMR

Proton T_2_ values in the muscle are elevated in different physiological and pathological scenarios such as necrosis and intracellular or interstitial edema (Arpan et al. 2013). Methodological efforts improve the precision of the T_2_ maps (Azzabou et al. 2015; Marty et al. 2016), but yet reliable interpretation of the data remains limited by the lack of specificity of the measure to the different underlying mechanism (Ploutz‐Snyder et al. 1997; Louie et al. 2009; Arpan et al. 2013). In our study, we observed no significant changes of the mean T_2_ or the mean T_2_w signal intensity during vascular draining nor vascular filling. Only three muscles showed significant alterations of the signal intensity or T_2_ in one of the two conditions as compared to the control condition. More sophisticated proton multicompartment T_2_ methodologies have been applied and have demonstrated a better sensitivity to extracellular volume variations. Using a localized proton ISIS‐CPMG sequence and a three‐sites two‐exchange model, authors provided quantitative information about histological tissues compartmentation and water exchange rates (Araujo et al. 2014). However, such a proton approach can be hampered by the signal originating from lipids. The bias due to fat signal contamination can be avoided using ^23^Na NMR, which represents an advantage for the characterization of cell viability in chronically affected muscle tissues.

Despite significant changes in ^1^H NMR parameters of some muscles during vascular volume changes, the ^23^Na NMR approach seems to be much more sensitive to monitor acute changes in extracellular volume fractions. This can have an influence on a variety of patient groups showing alterations in the tissue cellular volume fraction due to different causes. Hypertensive subjects for example demonstrated an altered Na^+^–K^+^ ATPase activity that also affects the biological response of skeletal muscle to physiological exercise (Dudley et al. 1990). Not only patients with refractory hypertension have an increased tissue Na^+^ which is detectable by ^23^Na‐NMR (Kopp et al. 2013), but also dystrophic muscle fibers are characterized by an extensive imbalance in the sodium homeostasis (Weber et al. 2012; Jakob et al. 2013). Sodium NMR has already measured changes in a variety of neuromuscular disorders including myotonic dystrophy, Duchenne muscular dystrophy, and channelopathies (Kushnir et al. 1997; Weber et al. 2006, 2011; Nagel et al. 2011). These diseases can express themselves with variable intensity in different muscles but the genetic defect spans over the entire musculature. Hence, one expects widespread distribution of perturbed sodium homeostasis or infiltration of collagenous connective tissue that contains high concentration of Na^+^.

In Duchenne patients, we would for example expect an increase in the total sodium content measured by the FID and increased TQF signal. The short $T_{2}^{*}$ might be longer and the long $T_{2}^{*}$ shorter. The prediction for the change of the global T_1_ is even more difficult. Thus, it would be very informative to study the impact of an increased membrane permeability on the proposed ^23^Na spectroscopic indices. Consequently, the proposed nonlocalized multiparametric ^23^Na approach could represent a powerful tool to describe the onset phase of diseases, revisit several aspects of their pathogenesis and ultimately monitor treatment in a novel noninvasive fashion.

### Methodological considerations

This study has a number of limitations. Problems arising with the ^23^Na‐NMR method are low SNR, strong sensitivity of the TQF signal intensity on B_0_ and B_1_ inhomogeneities (Fleysher et al. 2010). Depending on the organ, sensitivity of ^23^Na‐NMR is 3000–20,000 times smaller than the one of ^1^H (Madelin and Regatte 2013). For a sufficient SNR, long acquisition times are generally needed for sodium imaging. For example, at 3T, Nagel et al. (2011) acquired total sodium content (TSC) images of the lower leg in 8 min 20 sec and IR images in 10 min 20 sec with spatial resolution of 0.5 cm^3^. Stobbe and Beaulieu (Stobbe and Beaulieu 2005) developed an IR sequence for the human brain with a SNR value of 17 in an acquisition time of 11 min 6 sec and a spatial resolution of 0.25 cm^3^ at 4.7 T. TQF sequences suffer from even lower SNR (~1 order of magnitude lower than TSC sequences) and hence impose so far acquisition times incompatible with clinical use on patients. In their study about quantification of intracellular sodium in the human brain at 7 T, Fleysher et al. (2013) acquired a TQF volume covering the whole brain in 43 min 12 sec with spatial resolution of 0.8 × 0.8 × 1 cm^3^. Although promising, these sequences are not yet ready to be used in clinical trials or natural history studies, when reduced acquisition time and patient comfort represent important issues when designing NMR protocols.

Furthermore, concerns about absolute concentrations constitute the major limitation of this NMRS method for a direct application to longitudinal monitoring of Na^+^ during disease natural history studies or long‐term clinical trials. We report this approach as highly sensitive to detect subtle modifications of sodium content or biodistribution when assessment is performed within a single spectroscopic session. Since the NMR signal does not only depend on spin concentration, but also on coil quality factor and on $B_{1}^{+}$ and B_0_ field, all these parameters have to be taken into account for quantification. So far, phantoms with known Na^+^ concentrations have been used as external references to quantify the sodium content in vivo in several ^23^Na‐NMR imaging studies (Bansal et al. 2000; Constantinides et al. 2000; Nielles‐Vallespin et al. 2007; Weber et al. 2011). This idea could be extended to our protocol by using such external phantom containing a shifting reagent to resolve the peak of the phantom separately. But even then, the B_0_ and B_1_ field inhomogeneities issue has to be carefully addressed, which is not a trivial task.

Moreover, due to the nonlocalized approach, sodium pools from other tissue such as skin, tendons, and ligaments contributed to a certain extent to our results. Although its contribution is obviously smaller than the one of skeletal muscle given the size difference of the organs, its real impact on our findings is still to be studied. One possible solution would be to use localized spectroscopy methods. Nevertheless, classical schemes such as point resolved spatially localized spectroscopy (PRESS) (Bottomley 1987) or stimulated echo acquisition mode (STEAM) (Frahm et al. 1989) would lead to too long echo times by preventing the acquisition of fast decaying ^23^Na signals. Another localized method, namely ISIS (Ordidge et al. 1986), would be an alternative to acquire the sodium spectra including the short ^23^Na $T_{2}^{*}$ components in a defined region.

During the vascular filling and draining conditions, changes in the extracellular water volume fraction are induced. It has been previously reported that perfusion and $T_{2}^{*}$ reflecting oxygen concentration and capillary size increase considerably during reperfusion (Lebon et al. 1998) and return to basal values within few dozen seconds. It might be possible that changes in the extracellular compartmentation of fluid and sodium homeostasis in muscle cannot be resolved within the 5 min interval left to reach control condition.

However, internal unpublished data showed that the FID signal is back to the starting baseline value after 5 min once the cuff was released.

### Summary

As demonstrated in our study changes in extracellular volume fraction are traceable by using nonlocalized ^23^Na‐NMR spectroscopy. This approach allows the monitoring of total and intracellular sodium content, in addition to the distribution of sodium in a time compatible with clinical investigation. This biophysical information can be used to assess ion homeostasis and cell integrity in the skeletal muscle. In the context of neuromuscular disorders, these variables offers new options to investigate ion channel leakage, membrane integrity, or even fibrosis formation.

## Conflict of Interest

None declared.
